# The Interplay Between HIF‐1α and EZH2 in Lung Cancer and Dual‐Targeted Drug Therapy

**DOI:** 10.1002/advs.202303904

**Published:** 2023-12-10

**Authors:** Jianmin Wang, Cheng Yang, Huashen Xu, Xinyu Fan, Lina Jia, Yang Du, Shougeng Liu, Wenjing Wang, Jie Zhang, Yu Zhang, Xiaoxue Wang, Zhongbo Liu, Jie Bao, Songping Li, Jingyu Yang, Chunfu Wu, Jing Tang, Guoliang Chen, Lihui Wang

**Affiliations:** ^1^ School of Life Science and Biopharmaceutics Shenyang Pharmaceutical University Shenyang 110016 P. R. China; ^2^ Benxi Institute of Pharmaceutical Research Shenyang Pharmaceutical University Benxi 117004 P. R. China; ^3^ Key Laboratory of Structure‐Based Drug Design & Discovery of Ministry of Education School of Pharmaceutical Engineering Shenyang Pharmaceutical University Shenyang 110016 P. R. China; ^4^ Department of Pharmacy Shengjing Hospital of China Medical University Shenyang 110004 P. R. China; ^5^ School of Pharmacy Shenyang Pharmaceutical University Shenyang 110016 P. R. China; ^6^ Research Program in Systems Oncology Faculty of Medicine University of Helsinki Helsinki 00290 Finland

**Keywords:** dual‐targeted drugs, feedback regulation, EZH2, HIF‐1α, lung cancer

## Abstract

Interactions between oncogenic proteins contribute to the phenotype and drug resistance. Here, EZH2 (enhancer of zest homolog 2) is identified as a crucial factor that mediates HIF‐1 (hypoxia‐inducible factor) inhibitor resistance. Mechanistically, targeting HIF‐1 enhanced the activity of EZH2 through transcription activation of SUZ12 (suppressor of zest 12 protein homolog). Conversely, inhibiting EZH2 increased HIF‐1α transcription, but not the transcription of other HIF family members. Additionally, the negative feedback regulation between EZH2 and HIF‐1α is confirmed in lung cancer patient tissues and a database of cell lines. Moreover, molecular prediction showed that a newly screened dual‐target compound, DYB‐03, forms multiple hydrogen bonds with HIF‐1α and EZH2 to effectively inhibit the activity of both targets. Subsequent studies revealed that DYB‐03 could better inhibit migration, invasion, and angiogenesis of lung cancer cells and HUVECs in vitro and in vivo compared to single agent. DYB‐03 showed promising antitumor activity in a xenograft tumor model by promoting apoptosis and inhibiting angiogenesis, which could be almost abolished by the deletion of HIF‐1α and EZH2. Notably, DYB‐03 could reverse 2‐ME2 and GSK126‐resistance in lung cancer. These findings clarified the molecular mechanism of cross‐regulation of HIF‐1α and EZH2, and the potential of DYB‐03 for clinical combination target therapy.

## Graphical abstract

The negative feedback regulation between EZH2 and HIF‐1α is identified and confirmed in vitro and in vivo, which mediates multidrug resistance to HIF‐1 inhibitor and EZH2 inhibitor. DYB‐03 is a newly screened dual‐target compound that targeting both HIF‐1α and EZH2 and displays significant anti‐tumor activity in vitro and in vivo, indicating the potential for clinical combination target therapy.

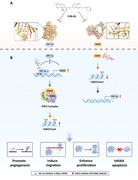

## 1 Introduction

Lung cancer is one of the most prevalent cancers globally and the leading cause of cancer‐related deaths, with an estimated 2 million new cases and 1.76 million deaths annually.^[^ ^1^ ^]^ Non‐small cell lung cancer (NSCLC) accounts for approximately 85% of all diagnoses based on the histopathological type.^[^ ^2^ ^]^ Treatment of NSCLC is exceptionally complex due to the presence of concurrent genomic alterations,^[^ ^3^ ^]^ metabolic reprogramming,^[^ ^4^ ^]^ and epigenetic abnormalities.^[^ ^5^ ^]^

Hypoxia is a hallmark feature of solid tumors such as NSCLC.^[^ ^6^ ^]^ Intratumoral hypoxia and the involvement of signaling molecules such as reactive oxygen species,^[^ ^7^ ^]^ cytokines,^[^ ^8^ ^]^ and growth factors^[^ ^9^ ^]^ results in highly activated HIF (hypoxia‐inducible factor). HIF is a transcription factor with two subunits, HIF‐1α (or its analogs HIF‐2α and HIF‐3α) and HIF‐1β. HIF‐1α is mainly located in the cytoplasm during normoxia, whereas during hypoxia, its levels increase and translocate into the nucleus.^[^ ^10^ ^]^ HIF‐1β, also known as aryl hydrocarbon receptor nuclear translocator (ARNT), is located in the nucleus and binds to HIF‐1α to activate hundreds of genes including those involved in angiogenesis, such as vascular endothelial growth factor (VEGF), platelet‐derived growth factor, and angiopoietin‐1.^[^ ^11^, ^12^ ^]^ Elevated HIF‐1α levels, however, are associated with poor prognosis in cancer patients.^[^ ^13^ ^]^ Thus, the therapeutic blockade of the HIF‐1 signaling pathway in cancer cells offers a promising strategy for developing anticancer drugs.^[^ ^14^ ^]^ Various HIF‐1 inhibitors such as 2‐ME2, 17‐AAG, EZN‐2208, and PX‐478 have been developed and studied in preclinical and clinical studies. Although 2‐ME2 can inhibit HIF‐1α protein synthesis and transcriptional activity, no objective response was observed in sixty patients with multiple myeloma when treated with 2‐ME2 in a phase II study. Due to the poor therapeutic efficacy of HIF‐1 inhibitors in clinical trials,^[^ ^15^, ^16^, ^17^, ^18^ ^]^ primary or acquired resistance to HIF‐1 inhibitors may exist. Therefore, it has become necessary to identify the mechanisms of resistance to HIF‐1 inhibitors and to seek improved combination therapy strategies.

Epigenetic modification^[^ ^19^ ^]^ is a biological process in which the DNA sequence remains constant, but the phenotype is changed since regulation is reversible and mainly consists of DNA methylation and histone modifications.^[^ ^20^ ^]^ Epigenetic dysregulation has been found to play an important role in the development, progression, and acquisition of therapeutic resistance in tumors, including solid and hematological malignancies. In addition to their potential in monotherapy treatments,^[^ ^21^ ^]^ epigenetic drugs may play an essential role in synergizing with other anticancer therapies^[^ ^22^ ^]^ or reversing drug resistance.^[^ ^23^, ^24^ ^]^ Preclinical evidence suggests that resistance to drugs targeting ErbB family receptor tyrosine kinases (RTK) is epigenetically driven, and thus can be reversed by epigenetic drugs. Many phase I and phase II studies have observed significant signs of delayed or reversed resistance, demonstrating the feasibility of combining histone deacetylase inhibitors (HDACi) and ErbB inhibitors in the treatment of patients with NSCLC.^[^ ^25^, ^26^ ^]^ These results suggest that reversal of epigenetic modifications is a desirable strategy to sensitize cancer cells to a given treatment regimen and to overcome resistance dynamically. However, the role of epigenetic regulation in the process of HIF‐1 inhibitor resistance remains unclear.

In the present study, we performed an epigenetic inhibitor library screen using HIF‐1 inhibitor‐resistant cells and identified an inhibitor of the histone methyltransferase EZH2 (enhancer of zest homolog 2), which sensitized the effect of HIF‐1 inhibitors. Furthermore, it was found that inhibiting HIF‐1α increased EZH2 enzyme activity. Conversely, HIF‐1α levels increased significantly when EZH2 was knocked down or treated with inhibitors. These results suggest that HIF‐1α and EZH2 may function as functionally complementary oncogenic signals, providing a novel therapeutic strategy to combine the two drugs in clinical trials. Meanwhile, we designed, synthesized, screened, and evaluated a series of HIF‐1α and EZH2 dual‐targeting compounds, among which DYB‐03 showed good antitumor activity by inhibiting NSCLC cell migration, invasion, angiogenesis, and reverse drug resistance in vitro and in vivo. Our work has uncovered the role of epigenetic enzymes in HIF‐1 inhibitor resistance and provides a pharmacological basis for the combination of HIF‐1 inhibitors and epigenetic enzyme inhibitors.

## 2 Experimental Section

### 2.1 Cell Lines and Cell Culture

Human NSCLC cell lines A549, NCI‐H460, and human umbilical vein endothelial cells (HUVEC) were purchased from ATCC (Manassas, VA, USA). U251‐HRE and U251‐PGL3 cells were provided by Giovanni Melillo (Developmental Therapeutic Program, National Cancer Institute, Frederick, MD). Cells were cultured in RPMI 1640 medium supplemented with 10% fetal bovine serum (FBS) (Gibco, Waltham, MA, USA) and 1% penicillin‐streptomycin (Gibco) at 37°C in a 5% CO_2_ incubator. Human embryonic kidney (HEK) 293T cells were cultured in Dulbecco's modified Eagle's medium (DMEM) (Gibco) supplemented with 10% FBS (Gibco) and 1% penicillin‐streptomycin (Gibco) at 37°C in a 5% CO_2_ incubator.

### 2.2 Establishment of Stable Cell Lines

HEK293T cells (5 × 10^6^ per plate) were seeded into 10 cm cell culture dishes and incubated overnight at 37°C and 5% CO_2_ for 12–18 h. When the cultured cells reached 80% confluence, HEK293T cells were co‐transfected with lentiviral expression constructs LentiCRISPRv2‐HIF‐1α sgRNA1 or LentiCRISPRv2‐EZH2 sgRNA2 (4 µg), viral envelope plasmid (pMD2.G, 4 µg), and viral packaging plasmid (psPAX2, 4 µg) using Lipofectamine 3000 (Invitrogen, California, USA) according to the manufacturer's protocols.^[^ ^27^ ^]^ Lentiviral expression constructs with scrambled shRNA were used as controls (specified as Scr). Viral supernatants were collected at 48–72 h post‐transfection, filtered through 0.45 µm filters and stored at −80°C until use. A549 or NCI‐H460 cells were seeded and incubated overnight before infection. The medium was replaced with a 1:2 dilution of viral supernatant supplemented with 10 µg mL^−1^ polybrene, set for 12 h, and replaced with an average growth medium. Stable scramble, HIF‐1α, and EZH2 knockout cell lines were selected using puromycin (2 µg ml^−1^, Sigma–Aldrich, Darmstadt, Germany) before the experiment used sgRNAs used in lentivirus expression vector construction for gene knockdown are shown in Supplementary Table 1.

### 2.3 CCK‐8 Assay

Cells (4 × 103per well) were seeded into a 96‐well plate and cultured at 37°C in a 5% CO_2_ incubator. After 24 h, different concentrations of 2‐ME2 were put into wells which ranged from 0.01 to 100 µm with a minimum of three technical replicates. After 72 h, 10 µl CCK‐8 (MCE, NY, USA) was added to each well and incubated for an additional 4 h at 37°C in a 5% CO_2_ incubator. Then, 100 µl DMSO was added to each well and mixed thoroughly. The optical density values were measured at 450 nm using a microplate reader (Molecular Devices, San Jose, CA, USA).

### 2.4 Western Blot Analysis

Cells were lysed with RIPA lysis buffer (Cell Signaling Technology, Danvers, MA, USA) containing a phosphatase inhibitor and protease inhibitor cocktail (Med Chem Express, Monmouth Junction, NJ, USA). Total protein was separated on sodium sulfate‐polyacrylamide gel electrophoresis (SDS‐PAGE) gels and then transferred onto polyvinylidene fluoride (PVDF) membranes. The membranes were blocked with 5% nonfat dried milk for 1 h at room temperature and incubated with the indicated primary antibody overnight at 4°C, followed by incubation with a secondary antibody for 1 h at room temperature. The protein bands were visualized with ECL detection reagents (Thermo Fisher Scientific, Waltham, MA, USA). Immunoblot was performed with anti‐HIF‐1α (BD, 610959, USA), SUZ12 (Proteintech, Cat. 20366‐1‐AP, China), EZH2 (Cell Signaling Technology, Cat. 5246), histone 3 lysine 27 trimethyl (H3K27me3) (Cell Signaling Technology, 9733S), H3 (Proteintech, Cat. 17168‐1‐AP), ARNT (Proteintech, Cat. 14105‐1‐AP), EPAS1 (Proteintech, Cat. 26422‐1‐AP), and EED (Proteintech, Cat.16818‐1‐AP) antibodies.

### 2.5 Co‐Immunoprecipitation (Co‐IP)

For Co‐IP assays, DSP (Thermo Fisher, Cat. 22585) was used to cross‐link interacting proteins. After the cross‐link, whole cell lysate was obtained using a RIPA buffer supplemented with PMSF, the protease inhibitor cocktail (Sigma–Aldrich, Cat. S8830), and phosphosite (Roche, Cat. 4906845001, Switzerland) followed by sonication and centrifugation at 12,000 rpm at 4°C for 10 min. IgG and A/G agarose (Thermo Fisher, Cat. 20421) were used to pre‐clean the cell lysate before IP with antibodies against EZH2 overnight at 4°C with gentle agitation. Then, 40 µl prewashed protein was added and incubated at a temperature of 4 h with gentle agitation. After extensive washing with an IP buffer twice and with PBS, the interacting proteins were eluted with SDS‐PAGE. Immunoblot was performed with anti‐HIF‐1α (BD, 610959), SUZ12 (Proteintech, Cat.20366‐1‐AP), EZH2 (Cell Signaling Technology, Cat.5246), and EED (Proteintech, Cat.16818‐1‐AP) antibodies.

### 2.6 RNA Isolation and qRT‐PCR Analysis

Total RNA was extracted using Trizol and transformed to cDNA using an All‐in‐One First‐Strand cDNA Synthesis Kit (Transgene, Beijing, China) following the manufacturer's instructions. qRT‐PCR analysis was performed by mixing 10 µl SYBR, 0.4 µM forward primer, 0.4 µM reverse primer, 2 µl cDNA, and 7.2 µl distilled water per sample with Top GreenTopR SuperMix (Transgene) according to the manufacturer's protocols. β‐actin was used as endogenous control. The primers for qRT‐PCR are listed in the Supplementary Table 2.

### 2.7 Immunohistochemical (IHC) Assay

Tumor samples acquired from mice bearing A549 cells were embedded in paraffin and antigen, and retrieval was performed. Following the blockade of endogenous peroxidase activity, samples were incubated with the primary antibodies of interest and the appropriate secondary antibodies and reacted with DAB detection reagents. As previously reported, the immunoreactive staining of proteins in tumor tissue was scored by applying a semiquantitative immunoreactive scoring (IRS) system. The median value of the score was chosen as the cut‐off criterion to dichotomize the results into high‐ and low‐expression subgroups.

### 2.8 Chromatin Immunoprecipitation (ChIP)

ChIP assays were performed using the Chromatin Immunoprecipitation Kit (Cell Signaling Technology) according to the manufacturer's protocol. A549 cells (1 × 10^7^) were briefly fixed with a cross‐linking solution and then collected. Samples were sonicated, and DNA was sheared to an average length of ≈250–450 bp. DNA–protein complexes were immunoprecipitated using 5 µg anti‐HIF antibodies or with polyclonal IgG control at 4°C overnight. Immunoprecipitated DNA was analyzed by quantitative PCR.

### 2.9 Luciferase Reporter Assays

HIF‐1 activity was assessed in U251‐HRE and U251‐PGL3 using a 96‐well format. Cells were rinsed twice with ice‐cold PBS, lysed with 200 µl of passive lysis buffer (Promega, Wisconsin, USA), and incubated at room temperature for 10 min. Lysates were centrifuged and stored at −80°C until assayed. Luciferase assays were conducted by pipetting 20 µl of cell lysate into each well of a 96‐well plate and analyzed immediately after adding the luciferase reagent (Promega) in a multi‐mode microplate reader (Centro LB960, Berthold, Germany).

### 2.10 Colony Formation Assay

To assess conformity in the action assay, cells were seeded into 6‐well plates at a concentration of 500 cells per well. Cells were treated with indicated compounds and cultured. The medium was refreshed every 3 days for 2 weeks. Colonies were fixed with methanol at room temperature for 30 min, and then stained with crystal violet (0.1% w/v in methanol) for 15 min and photographed.

### 2.11 Wound Healing Assay

A549 and H460 cells were seeded into a 24‐well plate. After the cell line reached 90% confluence, a 10 µl sterile pipette tip was used to create scratches in the cell monolayer. The dissociated cells were washed with PBS and replenished with RPMI 1640 medium without FBS. The cells were treated with different concentrations of DYB‐03. After 12 h or 24 h of incubation, cell migration was observed and imaged under a Nikon Ti2 microscope (Japan).

### 2.12 Transwell Assay

The effect of DYB‐03 on the invasion ability of A549 cells was assessed using Transwell (Corning Costar, USA). Matrigel was diluted with serum‐free culture medium spread evenly on the upper layer of Transwell chambers and was solidified at 37°C for 30 min before use. 500 µl complete culture medium was added to the lower chamber and 200 µl of cell suspension prepared from serum‐free culture medium was placed in the upper chamber, adjusting the cell density to approximately 2.5 × 105 ml^−1^. The cells were incubated in a hypoxia incubator chamber. The lower chamber was sectioned with PBS, fixed in 4% paraformaldehyde for 15 min, stained with crystal violet, and imaged using the Nikon Ti2 microscope.

### 2.13 CAM Assay

The in vivo chicken embryo CAM angiogenesis model was used as previously described. Leghorn fertilized eggs were incubated for 7 days at 37°C in the incubator, at which point a window was opened on the egg's shell to expose the CAM. The window was covered with tape, and the eggs were returned to the incubator. Different amounts of DYB‐03 (5, 10, and 20 µM) in a solution containing RPMI 1640 medium were applied to an area of 1 cm^2^ (restricted by a plastic ring) of the CAM on day 9 of embryo development. 48 h after treatment and subsequent incubation at 37°C, CAMs were fixed in situ, excised from the eggs, placed on slides, and left to air dry. Pictures were taken through a stereoscope equipped with a digital camera, and the total length of the vessels was measured using image analysis software (software; Scion Corporation, Frederick, MD). Assays for each test sample were conducted three times, with each experiment including 6 to 10 eggs per data point.

### 2.14 Tube Formation Assays

The 24‐well plates were pre‐loaded with 200 µl of matrix gel and then inoculated with HUVEC (3 × 10^5^ cells/well) after curing, and 10 ng mL^−1^ VEGF was added to each well. After 12 h, the cells were fixed and stained with calcein AM.^[^ ^28^ ^]^ Photographs were taken using an inverted fluorescence microscope (Nikon Ti2), and statistical analysis of parameters such as vessel formation length was performed using AngioTool (https://ccrod.cancer.gov/confluence/display/ROB2/Home).

### 2.15 In Vivo Matrigel Plug Assay

Matrigel (Corning, 356234) was frozen overnight at 4°C. C57/bl6 female mice (6‐8 weeks, Beijing Huafu Kang Laboratory Animal Technology Company, Beijing, China) were subcutaneously injected with 0.5 ml of Matrigel near the midline of the abdomen. The indicated concentrations of drugs and VEGF (final concentration 150 ng ml^−1^) and heparin (40 u ml^−1^) were added according to the grouping.^[^ ^29^ ^]^ After 10 days, the Matrigel was removed by execution, photographed, and recorded. Hemoglobin (Hb) concentrations were determined using hemoglobin cyanide (HiCN, Sigma), and the absorbance was measured at 530 nm for each group.

### 2.16 In Vivo Tumor Xenograft Animal Model

Female BALB/c nude mice (5‐6 weeks old) were purchased from the Beijing Huafu Kang Laboratory Animal Technology Company. Tumor volume was measured using a vernier caliper and calculated with the following formula: tumor volume (V) = width^2^× length × 0.52. For the A549 tumor xenografts experiment, 2 × 10^6^ A549 cells were injected subcutaneously into the right flank of the BALB/c nude mice. When the tumors reached ≈80–100 mm^3^, the mice were randomly divided into six groups for treatment: (1) vehicle; (2) 2‐ME2; (3) EPZ6438; (4) combination (2‐ME2 and EPZ6438); (5) DYB‐03 low‐dose (25 mg k^−1^g) group, and (6) high‐dose (50 mg k^−1^g) group. For the A549‐Double‐KD tumor xenografts experiment, 2 × 10^6^ A549‐Double‐KD cells were injected subcutaneously into the right flank of the BALB/c nude mice. When the tumors reached ≈80–100 mm^3^ the mice were randomly divided into two groups for treatment: (1) vehicle and (2) DYB‐03 high‐dose (50 mg k^−1^g) group. The tumor volumes and body weights were monitored weekly until the tumor volume reached 1000–1500 mm^3^. After the treatments, animals were anesthetized and tumors were excised.

### 2.17 Analysis of Clinical Specimens

Paraffin‐embedded clinical tissue specimens from 81 lung adenocarcinoma patients were obtained from Shanghai Outdo Biotech (Shanghai, China). Among the 81 patients, 30 were determined as stage III lung cancer, 48 were stage II lung cancer, and 3 patients were stage I lung cancer. The IHC staining of specimens was scored according to the intensity of dye color and the percentage of positive cells, and the average score was calculated in studied cases. A score higher than the average value was designated as high expression, and lower than average was designated as low expression.

### 2.18 Apoptosis Analysis

Drug‐resistant A549 and H460 cells were cultured until 70% confluence and exposed to 2‐M2, GSK126 or DYB‐03 at the indicated concentrations. After incubation for 48 h, cells were harvested by centrifugation and washed twice in PBS. Then stained the cells according to the FITC Annexin V Apoptosis Detection Kit II (BD Biosciences, USA). Control cells stained with Annexin‐V or PI alone were used to compensate for flow cytometric analysis. Viable cells were those with both Annexin‐V and PI‐double negative staining. Annexin‐V‐positive and PI‐negative cells were defined as early apoptotic cells, and Annexin‐V and PI‐double‐positive cells were defined as late apoptotic cells.

### 2.19 Pharmacokinetics Study In Vivo

SPF‐grade healthy Sprague‐Dawley Rat (6 females, 6 males, 8 weeks old, 180–200 g), were provided by Liaoning Changsheng Biotechnology Co., Ltd (Liaoning, China), license No. SCXK (Liao) 2015–0001. SD rats were housed under controlled environmental conditions and acclimatized for one week. 12 SD rats were randomly divided into two groups, which were administered with DYB‐03 via *iv* or *po* routes with the same dose of 20 mg k^−1^g. The blood sample was taken from the orbit vein at indicated time points (*iv*: 0.033, 0.12, 0.25, 0.5, 1, 2, 3, 4, 6, 8 and 10 h; *po*: 0.083, 0.17, 0.25, 0.5, 1, 2, 4, 6, 8 and 10 h), collected on Eppendorf containing anticoagulant EDTA‐K_2_, and centrifuged at 4500 rpm for 5 min. After protein precipitation of the plasma samples by methanol with internal standard (IS) B413, the chromatographic separation was carried out on a NanoChrom ChromCore C18 3µm, 3.0×50 mm column. The detection was performed on a triple quadrupole tandem mass spectrometer AB SCIEX Triple Quad^TM^ 4500MD by multiple reaction monitoring (MRM) mode via electrospray ionization (ESI) source on positive ion mode, with target quantitative ion pairs of m/z 480.2→128.0 for DYB‐03, 453.2→120.0 for B413 (IS). The calibration curve was linear over the range of 5–1000 ng mL^−1^ (r >0.99). The LLOQ was evaluated to be 5 ng mL ^−1^. All the pharmacokinetic parameters were obtained using Phoenix WinNonlin software.

### 2.20 Statistical Analysis

Data analysis was performed using the statistical program GraphPad Prism (GraphPad Prism, USA). Results are presented as mean ± SD unless otherwise indicated. Statistical analyses were performed using a two‐tailed Student's *t*‐test to derive the significance of the differences between the two groups. *p‐value* < 0.05 was considered statistically significant.

## 3 Results

### 3.1 EZH2 Mediates Resistance to HIF‐1 Inhibitors in Lung Cancer Cells

**Figure 1:**
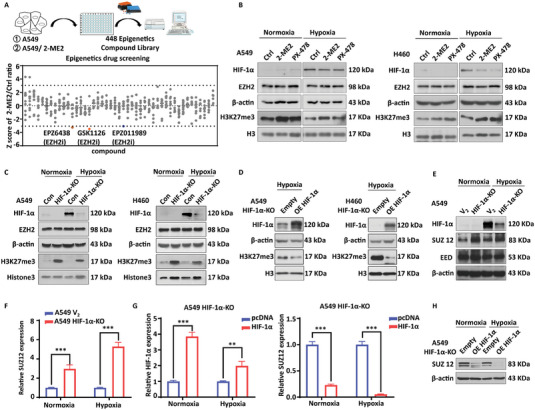
EZH2 mediates resistance to HIF‐1 inhibitors in lung cancer cells. A) Screening for HIF‐1 inhibitor‐resistant cells using an epigenetic compound library. B) HIF‐1 inhibitors promote high expression of EZH2 substrate H3K27me3 in A549 and H460 cell lines. C) Under normoxia and hypoxia, the knockdown of HIF‐1α promotes high expression of the EZH2 substrate H3K27me3. D) Rescuing HIF‐1α reduces H3K27me3 protein levels in A549 and H460 HIF‐1α knockout cells. E,F) Knockout of HIF‐1α promotes protein and mRNA levels of SUZ12. ^**^p < 0.01,^***^ *p* < 0.001, as compared with the V_2_ group. Rescuing HIF‐1α reduces SUZ12 mRNA levels G) and protein levels H) in HIF‐1α knockout cells. ^**^ *p* < 0.01, ^***^ *p* < 0.001, as compared with the pcDNA group.

To investigate the mechanism of resistance to HIF‐1 inhibitors, we constructed A549‐and H460‐resistant 2‐methoxy estradiol (A549/2‐ME2 and H460/2‐ME2 model) using a stepwise induction approach (Figure S1A, Supporting Information). We then examined the role of epigenetic enzymes in HIF‐1 inhibitor resistance using the Epigenetics Compound Library, including 57 Histone methyltransferase inhibitors (HMTi); 27 Histone Demethylase inhibitors; 66 Histone deacetylase inhibitors (HDACi); 26 Sirt inhibitors; 11 HATi; 83 bromodomain (BRD) inhibitors; 52 Janus Kinase inhibitors, and 126 others (**Figure** **1**A). The results showed that the resistant cells were more sensitive to the histone methyltransferase EZH2 inhibitors GSK126, EPZ6438, and EPZ011989 compared to the parental cells, suggesting that EZH2 may be involved in the process of resistance to HIF‐1 inhibitors. We then used the HIF‐1 inhibitors, including 2‐ME2 and PX‐478, to suppress HIF‐1α, and found that inhibition of HIF‐1α did not affect EZH2 protein expression levels, but did cause an increase in the level of the EZH2 substrate H3K27me3, indicating that inhibition of HIF‐1α results in an increased EZH2 enzyme activity (Figure 1B). Western blotting showed that in both cases of A549/2‐ME2 and H460/2‐ME2, EZH2 protein expression levels did not change, but an increase in the level of the EZH2 substrate H3K27me3 is observed under normoxic conditions (Figure S1B, Supporting Information). We then constructed A549 and H460 cells with a stable HIF‐1α knockout (Figure S1C, Supporting Information) and examined the expression of EZH2 and its substrate H3K27me3 under normoxic and hypoxic conditions. Similar to HIF‐1 inhibitor treatment, HIF‐1α knockout did not affect the expression of EZH2, but it did elevate the levels of the EZH2 substrate H3K27me3 (Figure 1C), whereas rescuing HIF‐1α inhibited the expression of H3K27me3 (Figure 1D).

The studies above show that the knockout of HIF‐1α results in an increase in EZH2 enzyme activity. We then performed IP assays to investigate whether this regulatory relationship was due to the physical interaction between HIF‐1α and EZH2. We discovered that HIF‐1α does not directly bind to EZH2 under hypoxic conditions (Figure S1D, Supporting Information). Furthermore, we investigated whether HIF‐1α knockdown affects the enzymatic activity of the EZH2 catalytic subunit by increasing the interactions between the members of the PRC2 (polycomb repressive complex 2) complex^[^ ^30^ ^]^ and showed that HIF‐1α knockout does not promote the binding of EZH2 to SUZ12 and EED (embryonic ectoderm development) (Figure S1E, Supporting Information). It has been reported that high expression of SUZ12 and EED in the PRC2 complex also increases the enzymatic activity of EZH2,^[^ ^31^ ^]^ so we examined the protein levels of SUZ12 and EED after HIF‐1α knockout under normoxic and hypoxic conditions. Our results indicated that HIF‐1α knockout significantly increased the protein levels of SUZ12 (Figure 1E). Similarly, knocking down HIF‐1α significantly increased SUZ12 mRNA levels, with a lesser effect on the other members (Figure 1F; Figure S1F, Supporting Information), whereas rescuing HIF‐1α inhibited the expression of SUZ12 (Figure 1G,H). Based on these results, the knockdown of HIF‐1α promotes the enzymatic activity of EZH2 by increasing the expression of SUZ12.

### 3.2 EZH2 Regulates the Expression of HIF‐1α in Lung Cancer

**Figure 2:**
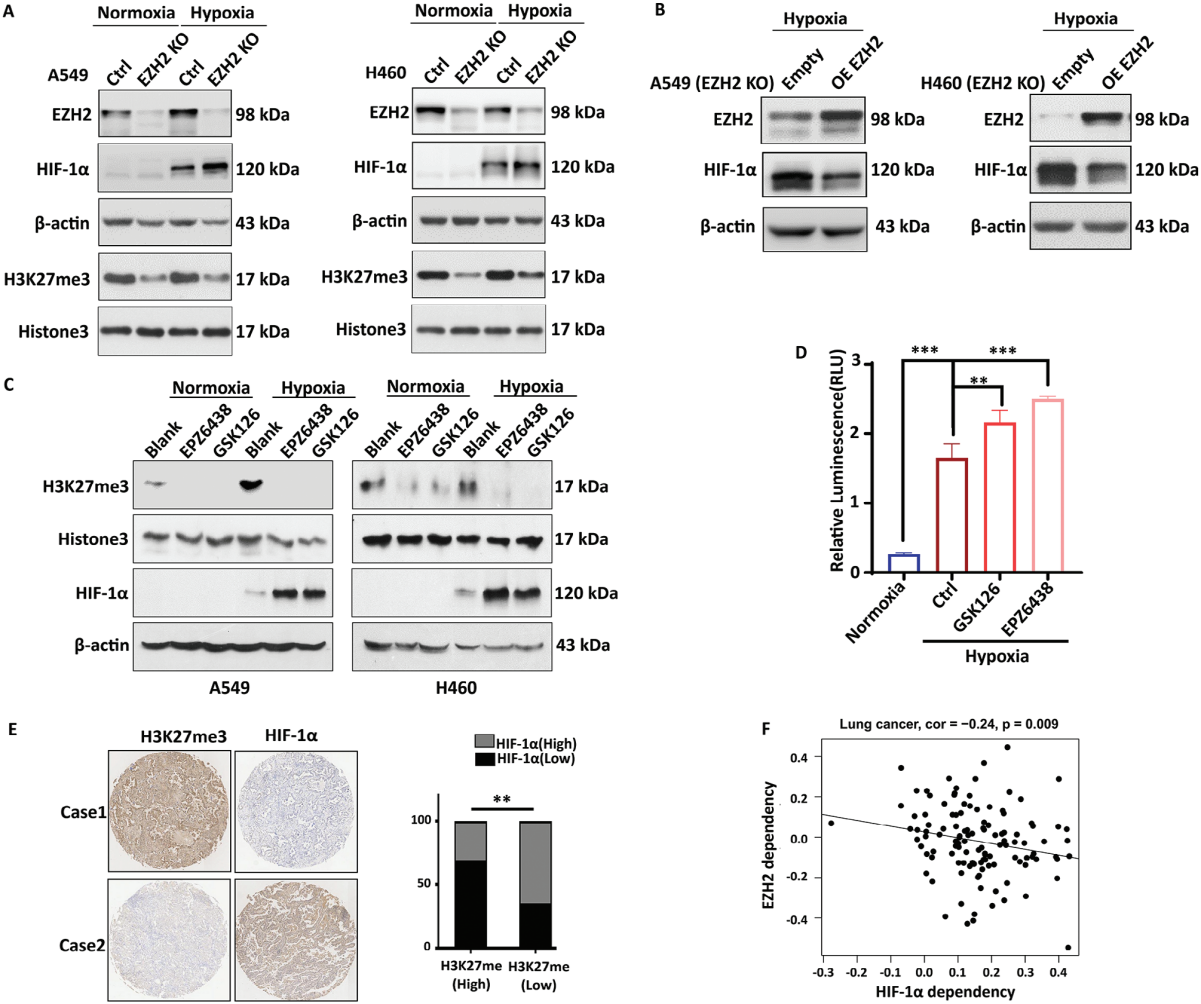
EZH2 regulates the expression of HIF‐1α in lung cancer. A) Knockout of EZH2 increases HIF‐1α protein levels under both normoxia and hypoxia. B) Rescuing EZH2 reduces HIF‐1α protein levels in EZH2 knockout cells. C) EZH2 inhibitors promote HIF‐1α protein expression. D) EZH2 inhibitors promote luciferase activity in U251 under hypoxic environments. E) IHC combined with Pearson correlation analysis was used to analyze the correlation between the expression of HIF‐1α and H3K27me3 in the tumor tissues of 81 patients. ^*^ *p* < 0.05, ^**^ *p* < 0.01, ^***^ *p* < 0.001, as compared with the control group or indicated group. F) The gene dependency scores for HIF‐1α and EZH2 were determined using the CRISPR knock‐out screens on 120 lung cancer cell lines (data retrieved from the DepMap portal https://depmap.org/portal/, version number: DepMap Public 23Q2+Score, Chronos). Lower dependency scores indicated stronger dependency on the genes for cancer survival.

Little has been reported on EZH2, an epigenetic enzyme that represses transcription, about its regulation of HIF‐1α transcription. Thus, we investigated the regulation of HIF‐1α by first knocking out EZH2 in A549 and H460 cells and then detecting the effect of gene manipulation of EZH2 on HIF‐1α expression. Our results showed that the knockout of EZH2 resulted in increased levels of HIF‐1α protein (**Figure** **2**A), which was reversed by the rescue of EZH2 (Figure 2B), indicating that EZH2 negatively regulates HIF‐1α expression. Similarly, using small molecule inhibitors of EZH2, such as GSK126 and EPZ6438 (Figure S2A, Supporting Information), resulted in significantly higher levels of HIF‐1α protein and promoted luciferase activity in U251 under hypoxic environments when compared to the control group (Figure 2C,D). Next, we investigated whether the knockdown of EZH2 also regulates other transcription factors of the HIF family. The results showed that the knockdown of EZH2 did not affect the expression of HIF‐2α and HIF‐1β (Figure S2B, Supporting Information). Next, we performed IHC to assess the relationship between HIF‐1α and H3K27me3 in NSCLC tumor tissue. Pearson correlation analysis showed a negative correlation between HIF‐1α and H3K27me3 in 81 tumor tissues (Figure 2E), which is consistent with our in vitro results. Furthermore, we found a negative correlation between the HIF‐1α and EZH2 dependency scores in lung cancer cell lines (Spearman cor =−0.24, *p* = 0.009), suggesting that lung cancer cells selectively rely on HIF‐1α or EZH2 for survival. This result is in line with the above experimental findings that the HIF‐1α‐EZH2 regulatory axis in lung cancer is not fulfilled via co‐expression. Meanwhile, identifying a direct EZH2‐HIF‐1α feedback loop in this study highlighted the essentiality of targeting dual directory regulation through combinatorial therapy, which would be otherwise underestimated by correlation analysis on gene dependency.

### 3.3 Synthesis, Screening, and Molecular Mechanisms of Dual‐Target Inhibitors

**Figure 3:**
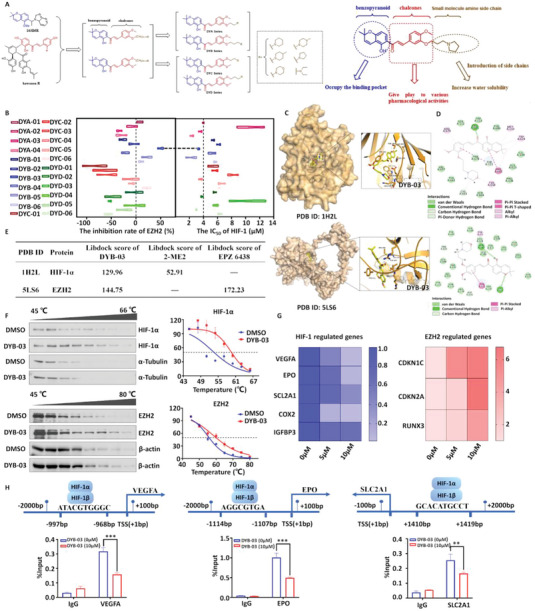
Synthesis, screening, and molecular mechanisms of dual‐target inhibitors. A) Ideas for the design of chalcone analogs of 2,2‐dimethylchromene. B) Luciferase assay to evaluate the HIF‐1 inhibitory activity of a series of compounds. DYB‐03 inhibits the activity of EZH2 and reduces the production of the substrate H3K27me3. C) The mode of action of DYB‐03 with the proteins HIF‐1α (above) and EZH2 (below), respectively. D) 2D view of DYB‐03 docked with HIF‐1α (above) and EZH2 (below), respectively. E) The LibDockScore of DYB‐03 with HIF‐1α and EZH2. F) CETSA shows that DYB‐03 binds to HIF‐1α and EZH2. G) DYB‐03 inhibits the expression of VEGFA, EPO, SLC2A1, COX2, and IGFBP3. DYB‐03 promotes the expression of CDKN2A, CDKN1C, and RUNX3. H) ChIP‐qPCR indicates that DYB‐03 inhibits HIF‐1α binding to HRE sites of promoter regions in EPO, SLC2A1, and VEGFA. ^**^p < 0.01,^***^p < 0.001, as compared with the DYB‐03 (0 µM) group.

Based on the findings above, HIF‐1α and EZH2 may play a functionally complementary carcinogenic role in cancer development and may account for single‐drug resistance and poor therapeutic efficacy. Natural products are an essential source for discovering and developing novel anticancer drugs.^[^ ^32^ ^]^ Since the natural product 103D5R has good HIF‐1 inhibitory activity with Kuwanon R,^[^ ^33^, ^34^ ^]^ we designed a series of chalcone analogs containing 2,2‐dimethyl chromene by referring to the chalcone structure of the natural product 103D5R and the chalcone structure of Kuwanon R, using the combination principles. Most small molecule inhibitors of EZH2 contain pyridone structures,^[^ ^35^ ^]^ which are readily metabolized in vivo, making the half‐life of currently developed inhibitors short in vivo and seriously compromising their anticancer efficacy. The 2,2‐dimethylchromene derivatives are a class of compounds based on structural modifications of natural products. Due to their relatively simple structure and moderate molecular size, they can be used as lead compounds for structural modification and screening of anticancer drugs. Based on the experimental validation above, we have obtained a total of 22 compounds in 4 series (DYA, DYB, DYC, and DYD) by changing the position and length of the carbon chain based on the previous development of HIF‐1 inhibitors while keeping the 2,2‐dimethylpyranones as the structural parent core (**Figure** **3**A).

Using a luciferase reporter gene assay, we first evaluated the inhibitory activity of four series of compounds against HIF‐1. The results showed that most compounds exhibited excellent HIF‐1 inhibitory activity, with seven compounds (DYA‐01, DYA‐03, DYA‐04, DYB‐03, DYB‐05, DYC‐04, and DYD‐05) with half maximal inhibitory concentration (IC_50_) less than 4 µM (Figure 3B). We then evaluated the inhibitory activity of the compound EZH2 by immunoblotting. As shown in Figure 3B, only DYB‐03 showed significant inhibition of the catalytic activity of EZH2, while the other compounds showed weak inhibition of EZH2. Based on the screening results above, we found that DYB‐03 displayed significant inhibitory activity against both HIF‐1 and EZH2. Next, we used BIOVIA Discovery Studio 2016 to predict the possible binding mode of a representative inhibitor (DYB‐03) in the binding site of HIF‐1α (PDB code: 1H2L) and EZH2 (PDB code: 5LS6) by molecular modeling. A diagram of the mode of action of DYB‐03 with the HIF‐1 protein is shown in Figure 3C. The oxygen atom in the benzopyran and the 4‐position substituted hydroxyl group form hydrogen bonds with THR196 and HIS199, respectively. The oxygen atom in the morpholine group forms a hydrogen bond with ASP201. The benzene ring creates a Pi‐Pi interaction with TYR102. The benzopyran is partially bound to the hydrophobic pocket. The diagram in Figure 3C illustrates the mode of action of DYB‐03 with the EZH2 receptor. The substituted keto carbonyl group on the benzopyran forms a hydrogen bond with ARG685. The oxygen atom in the morpholine group forms hydrogen bonds with ARG679 and PHE665, respectively. The benzene ring creates a Pi‐Pi interaction with TYR‐111. The benzene ring, as well as the side chain, are partially bound in hydrophobic pockets (Figure 3C, below). Additionally, the LibDockScore of DYB‐03 with HIF‐1α and EZH2 was higher than that of HIF‐1α inhibitor 2‐ME2, but lower than that of EZH2 inhibitor EPZ6438 (Figure 3D,E; Figure S3A, Supporting Information). To explore the interaction of DYB‐03 with HIF‐1α and EZH2, we used a cellular thermal shift assay (CETSA)^[^ ^36^ ^]^ for target confirmation. DYB‐03–treated cells showed a rightward shift in the melting curve compared to the DMSO control, indicating that DYB‐03 stabilized HIF‐1α and EZH2 (Figure 3F).

To investigate whether DYB‐03 could inhibit the activity of HIF‐1 and EZH2, we examined the mRNA levels of VEGF,^[^ ^37^ ^]^ EPO (erythropoietin),^[^ ^38^ ^]^ SLC2A1 (solute carrier family 2),^[^ ^39^ ^]^ COX2 (cyclooxygenase‐2),^[^ ^40^ ^]^ and IGFBP3 (insulin‐like growth factor binding protein 3),^[^ ^41^ ^]^ the downstream transcriptional genes of HIF‐1, after DYB‐03 treatment and found that they were all inhibited to varying degrees. EZH2 exerts its oncogenic effects by catalyzing the histone hypermethylation of promoter regions on tumor suppressor proteins, such as CDK (cell cycle protein‐dependent protein kinase). CDKN1C, CDKN2A,^[^ ^42^ ^]^ and RUNX3^[^ ^43^ ^]^ were differentially upregulated after DYB‐03 treatment, indicating that the transcriptional repressive activity of EZH2 was deregulated (Figure 3G). Furthermore, we wanted to investigate how DYB‐03 inhibited HIF‐1 activity. We first investigated whether HIF‐1α mRNA and protein levels were affected by DYB‐03 under normoxic and hypoxic conditions. The results showed that DYB‐03 treatment did not significantly reduce HIF‐1α mRNA and protein levels, indicating that DYB‐03 does not inhibit HIF‐1 activity by affecting HIF‐1α mRNA and protein levels (Figure S3B,C, Supporting Information). We hypothesized that DYB‐03 might inhibit HIF‐1 activity by affecting HIF‐1α binding to DNA. To test this, we used ChIP‐qPCR assay to explore whether DYB‐03 inhibited transcription by affecting the binding of HIF‐1α to HRE. The results showed that DYB‐03 treatment inhibited the binding of HIF‐1α to HRE, thereby suppressing the transcription of VEGFA, EPO, and SLC2A1 (Figure 3G,H). Together, the results showed that the dual‐target compound DYB‐03 has a strong binding affinity to HIF‐1α and EZH2 and inhibits their activity.

### 3.4 DYB‐03 Inhibits Migration and Invasion of Lung Cancer In Vitro and In Vivo

**Figure 4:**
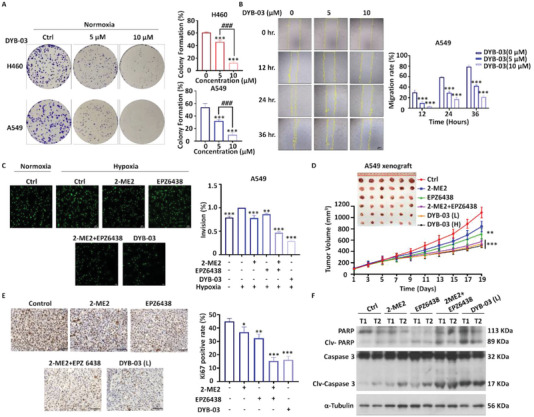
DYB‐03 inhibits migration and invasion of lung cancer in vitro and in vivo. A) The colony formation assay was assessed in DYB‐03–treated NSCLC cells. B) The migration ability was assessed in DYB‐03–treated A549 cells using wound healing assay. C) The invasion of A549 cells was evaluated by a Transwell assay. Cells treated with DYB‐03 or 2‐ME2 and EPZ6438 were seeded into the inner chamber. After 24 h incubation, the invasive cells were photographed. ^**^ *p* < 0.01, ^***^p < 0.001, as compared with the A549 (hypoxia) group. D) Tumor volumes were measured in A549 xenografts treated with 2‐ME2, EPZ6438, DYB‐03, or the combination of 2‐ME2 and EPZ6438. E) Representative IHC of Ki67 in A549 xenograft and statistical analysis of Ki67‐positive cells. F) The expression levels of apoptosis‐associated proteins in A549 xenografts. ^*^ *p* < 0.05, ^**^ *p* < 0.01, ^***^ *p* < 0.001, as compared with the control group.

It is established that HIF‐1 activation can promote cancer cell migration and invasion by activating the transcription of genes encoding the proteases MMP2, VEGF, and Angiopoietin‐like Protein 4.^[^ ^44^ ^]^ EZH2 contributes to cancer development by regulating cell cycle progression and promoting cell invasion in many cancers.^[^ ^45^ ^]^ To investigate the effect of DYB‐03 on the proliferation of NSCLC, we performed colony formation assays. Our data showed that DYB‐03 inhibited the proliferation ability of A549 and H460 cells in a concentration‐dependent manner (**Figure** **4**A). Wound healing assay revealed that DYB‐03 treatment, at a nontoxic concentration (Figure S4A, Supporting Information), could lead to the suppression of cell migration (Figure 4B and Figure S4B, Supporting Information). Enhancing the invasive ability of epithelial cells is the initial step in tumor metastasis. Thus, we investigated the effect of DYB‐03 on the invasive ability of epithelial cells using Transwell analysis. Our data showed that the number of A549 invasions increased under hypoxic conditions and that DYB‐03 significantly inhibited cell invasion with an effect comparable to that of the two single‐target inhibitors in combination (Figure 4C). Based on the antitumor activity demonstrated by DYB‐03 in vitro, we further investigated the in vivo efficacy of DYB‐03 in A549 xenograft models. The results showed that the combination of the two drugs, as well as DYB‐03, significantly inhibited the growth rate of A549 xenograft tumors and achieved a tumor inhibition rate of approximately 58% compared to the poor efficacy demonstrated by the single drug, but there was no significant difference between the high and low doses (Figure 4D; Figure S4C, Supporting Information). In addition, DYB‐03 did not cause any toxic effects based on body weight observations (Figure S4D,E, Supporting Information). The antitumor effect was further confirmed by staining for the cell proliferation marker, Ki67. As shown in Figure 4E, Ki67 expression was lower in the DYB‐03 treated group than in the control group. In addition, we examined the expression of apoptotic proteins in the tumors by western blotting. The results showed that the apoptosis‐related proteins PARP (poly ADP‐ribose polymerase) and caspase3 were significantly up‐regulated in the DYB‐03–treated group, indicating that DYB‐03 can exert its tumor‐suppressive effect by activating the expression of apoptosis‐related proteins (Figure 4F). Taken together, the totality of evidence indicates that DYB‐03 has excellent antitumor activity in vitro and in vivo.

### 3.5 DYB‐03 Inhibits Angiogenesis In Vitro and In Vivo

**Figure 5:**
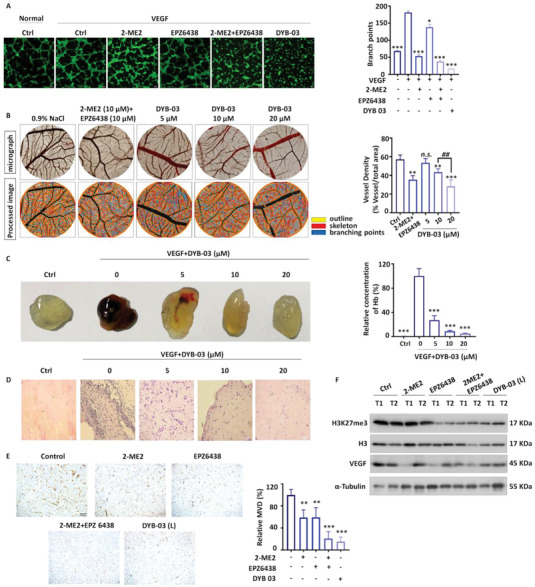
DYB‐03 inhibits angiogenesis in vitro and in vivo. A) Tube formation of HUVEC cells on Matrigel. Cells were treated with DYB‐03 (5 µM) or 2‐ME2 and EPZ6438 for 12 h. B) The newly formed blood vessels on the chick embryo CAM after being treated with DYB‐03 (0, 5, 10, and 20 µm) for 48 h. C) Matrigel plug assays were applied to evaluate the in vivo inhibition of DYB‐03 in anti‐angiogenesis. Matrigel with or without VEGF (300 ng mL^−1^) was subcutaneously injected into C57/BL6 mice. D) The HE stain of Matrigel. E) Representative IHC of CD31 in A549 xenograft and statistical analysis of CD31‐positive cells. ^*^p < 0.05, ^**^p < 0.01, compared with the control group. F) The expression levels of substrate proteins of HIF‐1 and EZH2 in A549 xenograft. ^*^ *p* < 0.05, ^**^ *p* < 0.01, ^***^p < 0.001, compared with the control (VEGF) group.

The angiogenic effect is mainly due to the production of VEGF, so we sought to investigate whether DYB‐03 could disrupt VEGF‐induced angiogenesis in vitro and in vivo.^[^ ^46^ ^]^ We performed a tube formation assay, a chicken embryo CAM assay, and a Matrigel plug assay in vivo. Despite the complexity of the angiogenesis process, tube formation of endothelial cells is one of the key steps. In the tube formation assay, HUVEC cells formed slender and tubular solid structures in the presence of VEGF stimulation. Treatment with DYB‐03 significantly inhibited the formation of tubular structures (**Figure** **5**A and Figure S5A, Supporting Information). In the CAM assay, treatment with DYB‐03 blocked neovascularization, leading to a reduction in microvascular density (Figure 5B). In addition, Matrigel mixed with VEGF was implanted subcutaneously in C57/BL6, and VEGF (300 ng mL^−1^) in Matrigel plugs (shown in dark red) significantly stimulated neovascularization compared to controls (no VEGF). In contrast, the number of angiogenic vessels was reduced considerably in Matrigel from mice treated with DYB‐03 for 14 days, and this result was consistent with HE staining (Figure 5C,D). These results suggest that DYB‐03 has antiangiogenic effects in vivo and in vitro. To understand whether the inhibitory effect of DYB‐03 could be attributed to angiogenic disruption, histological analysis of tumor tissue was performed to detect the expression of CD31, a marker of microvascular density.^[^ ^47^ ^]^ CD31 was significantly inhibited in the DYB‐03–treated group compared to the control group, suggesting that DYB‐03 partially inhibited tumor growth through its antiangiogenic effect (Figure 5E). It was also demonstrated that DYB‐03 could exert a dual‐targeting effect in vivo, inhibiting the expression of VEGF and H3K27me3 (Figure 5F). In summary, DYB‐03 has superior antiangiogenic capacity.

### 3.6 DYB‐03 Executes Antitumor Function by Targeting HIF‐1 and EZH2

**Figure 6:**
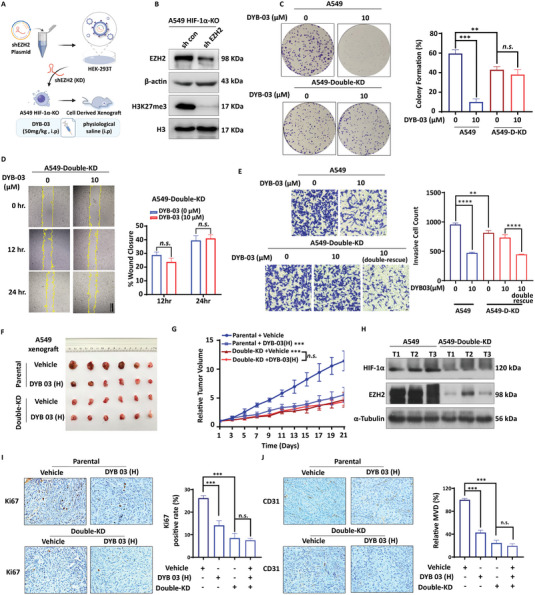
DYB‐03 executes antitumor function by targeting HIF‐1 and EZH2. A) Construction of a double knockdown cell line of shEZH2 on A549 HIF‐1α cells. B) The protein level of EZH2 in A549 double knockdown cells. C) Colony formation assay was performed in DYB‐03–treated A549 double knockdown cells. D) The migration ability was assessed in DYB‐03–treated A549 double knockdown cells by scratch‐wound healing assay. E) The invasion of A549 double knockdown cells was evaluated by a Transwell assay. After 24 h incubation, the invasive cells were photographed. F) Tumor G) and tumor volumes were measured in A549 double knockdown cells xenografts treated with DYB‐03. H) The expression of HIF‐1α and EZH2 protein in A549 double knockdown cell xenografts. Representative IHC of Ki67 (I) and CD31 J) in A549 xenograft and statistical analysis of Ki67‐positive and CD31‐positive cells. ^***^p < 0.001, compared with the control group.

The studies above suggested that DYB‐03 exerts its antitumor effects by inhibiting migration and invasion of NSCLC and angiogenesis in vitro and in vivo. It is worth further investigating whether DYB‐03 exerts these effects by acting on HIF‐1 and EZH2. We established a stable EZH2/HIF‐1α double knockdown cell line (**Figure** **6**A,B; Figure S6A, Supporting Information). As expected, compared with parental cells, EZH2/HIF‐1α double knockdown cells partially lost the abilities in proliferation and migration, indicating the role of both proteins in tumorigenesis (Figure 6C,E). Furthermore, colony formation and a wound‐healing assay showed that DYB‐03 had no significant effect on the proliferation and migration of the double knockdown cells compared to the control (Figure 6C,D). Similarly, Transwell assays showed that the ability of DYB‐03 to inhibit cell migration was absent, but when we reinfused the cells with HIF‐1α and EZH2, this inhibition was reversed, suggesting that DYB‐03 acts on HIF‐1 and EZH2 (Figure 6E; Figure S6B, Supporting Information). We then confirmed the target specificity of DYB‐03 in vivo and showed that tumor growth was slowed by double knockdown of HIF‐1α and EZH2 in A549, similar to the inhibitory effect of DYB‐03 on parental cells. Additionally, administration of DYB‐03 did not have a significant inhibitory effect on tumor growth in A549 double knockdown cells, indicating that DYB‐03 exerts antitumor activity by targeting HIF‐1α and EZH2 (Figure 6F,G and Figure S6C, Supporting Information). Meanwhile, western blotting verified the protein stability of the A549 double‐knockout cells in vivo (Figure 6H). Histological analysis of tumor tissue was also performed to detect the expression of Ki67 and CD31. As shown in Figure 6I,J, Ki67 and CD31 expression was much lower in the double knockdown tumor tissues than in the parental tumor tissues. DYB‐03 administration showed a significant antiproliferative effect and antiangiogenic effect in the parental group, while a minor effect in the A549 double knockdown group. The results above suggested that DYB‐03 also exerts its tumor suppressive effect in vivo by acting on HIF‐1 and EZH2.

### 3.7 DYB‐03 Reverses Drug Resistance of Lung Cancer and Its Pharmacokinetics

**Figure 7:**
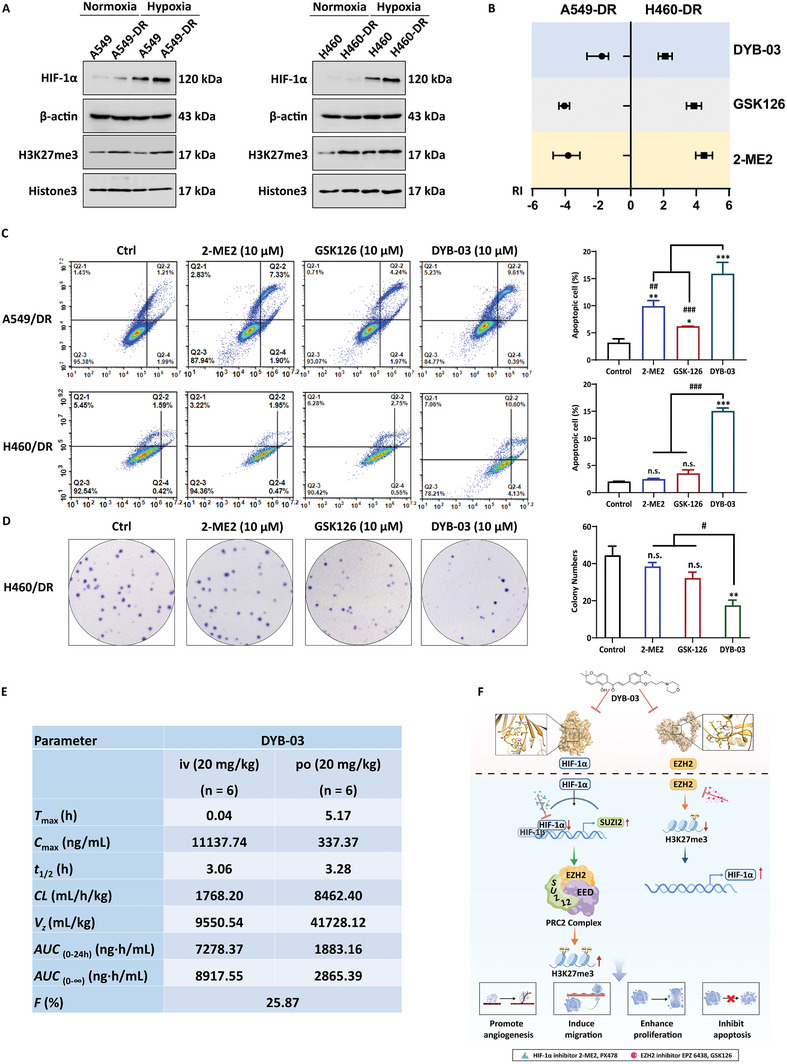
DYB‐03′s reversal effect on 2‐ME2‐ and GSK126‐resistance and pharmacokinetics in experimental animals. A549‐DR and H460‐DR cells were both 2‐ME2‐ and GSK‐126‐resistant lung cancer cell lines. A) Under normoxia, high expression of HIF‐1α and the EZH2 substrate H3K27me3 in A549‐DR and H460‐DR cells. B) The resistance ratio (RI) of the IC_50_ in drug‐resistant cells compared to the IC_50_ in parental cells was calculated to determine the efficacy of DYB‐03. C) The Annexin V‐FITC/PI staining assay was assessed in A549‐DR and H460‐DR cells after DYB‐03 treatment. D) The colony formation assay was assessed in A549‐DR and H460‐DR cells after DYB‐03 treatment. ***p < 0.001, compared with the control group. ##*p* < 0.01, ###*p* < 0.001, compared with the DYB‐03 group. E) ^a^Species: SD rats; after administration, blood samples were collected at different times (*iv*: 0.033, 0.12, 0.25, 0.5, 1, 2, 3, 4, 6, 8, and 10 h; *po*: 0.083, 0.17, 0.25, 0.5, 1, 2, 4, 6, 8, 10, 14, 18, and 24 h); compounds formulated in 0.5% CMC‐Na solution were administered orally at the indicated doses; the combination of compounds in a ratio of 5% DMSO, 5% Tween‐80, and 90% normal saline was chosen for the intravenous injection formulation. F) The interplay between HIF‐1α and EZH2 in lung cancer and dual‐targeted drug therapy.

Based on the above results, the HIF‐1α‐EZH2 regulatory axis mediated multidrug resistance in lung cancer cells. The reversal effect of DYB‐03 on multidrug resistance was estimated in the following. 2‐ME2‐resistant and GSK‐126‐resistant lung cancer cell lines were developed across different lung cancer cell lines (H460 and A549) using a stepwise induction approach. Compared to parental cells, the level of HIF‐1α was increased in A549/DR and H460/DR cells. Similarly, the EZH2 substrate H3K27me3, but not EZH2, was also overexpressed in dual drug‐resistant cell lines (**Figure** **7**A). These results demonstrated that an increased EZH2 enzyme activity and HIF‐1α protein expression results in multidrug resistance to HIF‐1 inhibitor and EZH2 inhibitor. Next, we examined the cytotoxic effect of DYB‐03 on dual cell lines comprising both drug‐resistance by CCK‐8 assay (Figure 7B). The resistance ratio (RI) of the IC_50_ in drug‐resistant cells compared to the IC_50_ in parental cells was calculated to determine the efficacy of DYB‐03. A549/DR and H460/DR cells comprising both drug‐resistance were resistant to 2‐ME2 and GSK126 with a resistance ratio ≈4, whereas the resistance ratio for DYB‐03 was 1.9–2.0, indicating that dual cell lines comprising both 2‐ME2‐ and GSK126‐resistance were much more sensitive to DYB‐03.

The apoptosis ratios of 2‐ME2‐ and GSK126‐resistant cells treated with **DYB‐03** were then quantified by the Annexin V‐FITC/PI staining assay (Figure 7C). After 48 h exposure, **DYB‐03** led to a significant increase in the apoptotic cell population in A549‐DR cells (13.95%) and H460‐DR cells (15.07%). In contrast, the percentage of apoptotic cells after 2‐ME2 or GSK126 treatment at the same concentration in A549‐DR cells and H460‐DR cells was much lower than that after **DYB‐03** treatment. These results suggested that DYB‐03 was more effective in inducing drug‐resistant cell apoptosis compared to that of the two single‐target inhibitors. We also evaluated the long‐term growth‐inhibitory effect of DYB‐03 on 2‐ME2‐ and GSK126‐resistant cells by colony‐forming assay (Figure 7D). Compared to 2‐ME2 or GSK126, DYB‐03 exhibited more potent inhibitory activities in H460/DR cells. Taken together, these data suggested that DYB‐03 could significantly reduce aberrant cell proliferation and hinder cell colony growth in multidrug‐resistant cells. These data suggested that the dual‐target compound DYB‐03 displays favorable anti‐MDR activity and overcomes 2‐ME2‐ and GSK126‐ resistance, indicating the clinical potential of DYB‐03 in the treatment of multidrug‐resistant NSCLC.

To clearly understand the druggable characteristics of DYB‐03, the pharmacokinetic study of DYB‐03 was done. After oral (po) and intravenous (iv) administration at the same dose, blood levels were analyzed for 12 or 24 h, and corresponding pharmacokinetic parameters were listed in Figure 7E. All in all, DYB‐03′s *iv* routes had higher AUC_(0‐10 h)_ and AUC_(0‐∞)_, smaller clearance (CL), and lower apparent volume of distribution, indicating that DYB‐03 penetrated the tissue slowly and contributed to a higher AUC. DYB‐03's *po* routes could distribute wider in vivo and then contribute to a decrease in AUC. However, the half‐lives (*t*_1/2_) of the two routes were similar. The analytical data suggested that *iv* routes could be a suitable administration mode for DYB‐03, which followed a triphasic model.

## 4 Discussion

Hypoxia, a hallmark of solid tumors, and high expression of HIF‐1α in human cancers are associated with poor patient prognosis and response to radiotherapy.^[^ ^46^ ^]^ As a result, HIF‐1α is considered to be a high potential therapeutic target, and blocking HIF‐1α may be a promising strategy for the treatment of cancer. Various inhibitors against HIF‐1 such as 2‐ME2, PX‐478, and 17‐AAG are currently in phase II clinical trials.^[^ ^48^ ^]^ However, their poor specificity and mediocre efficacy in treating tumors suggest that it is important to investigate the causes of resistance to HIF‐1 inhibitors.^[^ ^15^, ^17^, ^49^ ^]^ Epigenetic enzyme dysregulation has been reported to be involved in the resistance process of several inhibitors.^[^ ^50^ ^]^ Tumor re‐sensitization to drugs can be activated by pharmacological inhibition of DNA and histone epigenetic modifications, and some epigenetic modification drugs have been approved in clinical trials as modulators of drug resistance reversal, with some drug combinations showing excellent clinical results.^[^ ^51^ ^]^ The binding of BRD4 (bromodomain‐containing protein 4) to the promoter region of the RTK gene can be prevented by the use of BET (bromodomain and extraterminal domain) inhibitors, addressing the upregulation of RTK transcription upon PI3K (phosphatidylinositol 3 kinase) inhibition and thus overcoming PI3K inhibitor resistance.^[^ ^52^ ^]^ Our research shows that the enzyme catalytic subunit EZH2 activity in the PRC2 complex is enhanced during HIF‐1 inhibitor resistance and that the combination of HIF‐1 inhibitor and EZH2 inhibitor exhibits better antitumor activity than single agent. Our findings support the dysregulation of epigenetic enzymes behind HIF‐1 inhibitor resistance and suggest a more attractive dual‐targeted treatment strategy.

In various cancers, HIF‐1α acts as a transcriptional activator and promotes the expression of genes for proliferation, metastasis, invasion, and angiogenesis by recruiting co‐activators such as p300/CBP acetyltransferases.^[^ ^53^, ^54^ ^]^ In contrast, reports of HIF‐1α as a transcriptional repressor are less common. M. Koshiji et al showed that in a hypoxic environment, HIF‐1α replaces the Sp1‐bound transcriptional activator Myc and represses MutSalpha expression, leading to destabilization of the genome of colon cancer cells.^[^ ^55^ ^]^ Similarly, Hwang‐Verslues et al found that under hypoxic conditions, HIF‐1α represses DSG2 (desmoglein‐2) transcription by recruiting EZH2 at the DSG2 promoter region, suggesting an oncogenic role for HIF‐1α in promoting breast cancer progression.^[^ ^56^ ^]^ In our study, HIF‐1α transcriptionally repressed the expression of SUZ12 and thereby inhibited the enzymatic activity of the PRC2 complex. However, more biological studies are needed to further understand the specific mechanism by which HIF‐1α achieves transcriptional repression of SUZ12.

Multi‐target therapy has been widely applied in clinics for the treatment of various cancers.^[^ ^57^, ^58^, ^59^, ^60^ ^]^ As a dual‐targeted inhibitor of HIF‐1α and EZH2, DYB‐03 binds well to the dual targets and exhibits potent inhibitory activity. HIF‐1 inhibitors can usually affect the following links:^[^ ^61^ ^]^in vivo HIF‐1α mRNA expression, HIF‐1α protein levels, HIF‐1α/HIF‐1β dimerization, HIF‐1α‐DNA binding, or HIF‐1α transcriptional activity. We found that DYB‐03 does not affect the HIF‐1α protein or mRNA levels, but rather inhibits HIF‐1 activity by disrupting the binding of HIF‐1α to the downstream gene promoter region. At the same time, DYB‐03 binds the active pocket of EZH2, inhibiting its methylation of substrates and promoting normal cell cycle regulation. Our results showed that the combination of 2‐ME2 and EPZ6438 or DYB‐03 alone showed better inhibition of tumor cell migration, invasion, and angiogenesis compared with single agents. Combination therapy with 2‐ME2 and EPZ6438 demonstrated promising antitumor activity in an in vivo A549 xenograft tumor model, suggesting that dual targeting of HIF‐1α and EZH2 is an emerging and attractive therapeutic approach. Again, the dual‐targeted compound DYB‐03 was very effective and well tolerated. Our study revealed the molecular mechanism of interrelation between HIF‐1α and EZH2 and provides a pharmacological basis for the dual targeting of HIF‐1α and EZH2, and the dual‐targeting compound DYB‐03 may be a promising candidate for the treatment of various cancer, such as lung cancer.

It is worth noting that the relationship pattern between EZH2 and HIF‐1α is not unique in cancer. For example, BRD4 promoted the degradation of MYC. However, MYC inhibited the HAT activity of BRD4.^[^ ^62^ ^]^ FOXM1 is also reported to interact directly with MATA1, and they negatively regulate each other.^[^ ^63^ ^]^ This shows that this special way of mutual regulation may be one of the characteristics of tumors and an unknown factor affecting cancer treatment. In this article, we realize that EZH2 and HIF‐1α, as important histone methyltransferases and transcription factors, their expressions play an important role in the normal physiological activities of human beings and their balance can determine the fate of cells. This means that the out‐of‐control expression of any molecule will affect the expression of another molecule, leading to the breakdown of the balance. At this time, the use of a single inhibitor will aggravate this imbalance and lead to treatment failure. This is precisely the advantage of dual‐target drugs.

Taken together, our study demonstrated that the epigenetic modifying enzyme EZH2 is involved in the process of HIF‐1α inhibitor resistance. Inhibition of HIF‐1α causes loss of SUZ12 transcriptional repression in the PRC2 complex, resulting in increased EZH2 enzyme activity. Meanwhile, EZH2 functions as a transcriptional repressor of HIF‐1α. HIF‐1α and EZH2 interact to form a negative feedback loop that promotes cancer development. The dual‐targeted inhibitor DYB‐03 binds excellently to EZH2 and HIF‐1α to exert antitumor effects in vitro and in vivo (Figure 7F). These results provide a pharmacological rationale for the dual targeting of HIF‐1α and EZH2.

## Conflict of Interest

The authors declare no conflict of interest.

## Supporting information

Supporting Information

## Data Availability

The data that support the findings of this study are available from the corresponding author upon reasonable request.
