# Supplementary material for: The Interplay Between HIF‐1α and EZH2 in Lung Cancer and Dual‐Targeted Drug Therapy
Source: Adv Sci (Weinh). 2023 Dec 10;11(7):2303904. doi: 10.1002/advs.202303904 (PMC10870044; doi:10.1002/advs.202303904)
Supplement: Supplementary file 1 — Supporting Information [file ADVS-11-2303904-s001.pdf]

ADVANCED SCIENCE
Open Access

Supporting Information

for *Adv. Sci.*, DOI 10.1002/advs.202303904

The Interplay Between HIF-1α and EZH2 in Lung Cancer and Dual-Targeted Drug Therapy

Jianmin Wang, Cheng Yang, Huashen Xu, Xinyu Fan, Lina Jia, Yang Du, Shougeng Liu, Wenjing Wang, Jie Zhang, Yu Zhang, Xiaoxue Wang, Zhongbo Liu, Jie Bao, Songping Li, Jingyu Yang, Chunfu Wu, Jing Tang, Guoliang Chen and Lihui Wang**

Supplementary Information

The interplay between HIF-1α and EZH2 in lung cancer and dual-targeted drug therapy

Supplementary Table 1 The sequence of sgRNAs used in lentivirus expression vector construction for gene knockdown

	Primer	Sequence (5' to 3')
HIF-1α sgRNA	Forward	CACC GCCTCACACGCAAATAGCTGA
	Reverse	AAAC TCAGCTATTTGCGTGTGAGGC
EZH2 sgRNA	Forward	CACC GCAATGAGCTCACAGAAGTC
	Reverse	AAAC GACTTCTGTGAGCTCATTGC

Supplementary Table 2 The sequence of primers for qRT-PCR

	Primer	Sequence (5' to 3')
chip-qPCR	VEGFA-F	GTAGGTTTGAATCATCACGCAGG
	VEGFA-R	GCACCAAGTTTGTGGAGCTGA
	EPO-F	GTCGCAAGGCATCAGA
	EPO-R	CTTGTCCTAGCGGGTT
	SCL2A1-F	AGTAAGGCACTGGTCTAGAGGA
	SCL2A1-R	GTCTCAAACTCCTGGGCTCAAG
HIF-1 regulated gene	COX2-F	GGCGCAGTTTATGTTGTCTGT
	COX2-R	CAAGACAGATCATAAGCGAGGA
	IGFBP3-F	CATCAAGAAAGGGCATGCTAAA
	IGFBP3-R	GAGGAGAAGTTCTGGGTATCTG
	EPO-F	ATGTGGATAAAGCCGTCAGTG
	EPO-R	AAAGTGTCAGCAGTGATTGTTC
	VEGFA-F	AGGAGGGCAGAATCATCACG
	VEGFA-R	CAAGGCCCACAGGGATTTTCT
	SCL2A1-F	GCAAGTCCTTTGAGATGCTGATCC
	SCL2A1-R	GCCGACTCTCTTCCTTCATCTCC
EZH2 regulated gene	RUNX3-F	CAACTTCCTCTGCTCCGTGCTG
	RUNX3-R	TTCTCGTCATTGCCTGCCATCAC
	CDKN1C-F	CAGAACCGCTGGGATTACGACTTC
	CDKN1C-R	TCGCTGTCCACTTCGGTCCAC
	CDKN2A-F	GGCCGATCCAGGTCATGATGATG
	CDKN2A-R	ACCACCAGCGTGTCCAGGAAG
PRC2 complex	RBBP4-F	CGACGCAGTGGAAGAACGAGTG
	RBBP4-R	TCCCTTCTGGTCTGGTTACATCTGG

	RBBP7-F	CTGTGGAGGAGCGTGTCATCAATG
	RBBP7-R	GGAAGCCACTGAACGGTAAGACTG
	AEBP2-F	CACACTTCAGTCAGCAGAACTCCTC
	AEBP2-R	GCCGTGGTAATGAGCGTCGTC
	SUZ12-F	GTTACCCTGGAAGTCCTGCTTGTG
	SUZ12-R	ACCTGTGGGAACTTGCCTTATTGG
	EED-F	TGAGCAGTGACGAGAACAGCAATC
	EED-R	TTGGCGTGTTTGTAGGTGTATCAGG
β-actin	ACTIN-F	TCGTGCGTGACATTAAGG
	ACTIN-R	AAGGAAGGCTGGAAGAGT
HIF-1α	HIF-1α-F	CGCAAGTCCTCAAAGCACAGTT
	HIF-1α-R	GGCAGTGGTAGTGGTGGCATT
HIF-1β	HIF-1β-F	TGCTGCTGCCTACCCTAGTCTC
	HIF-1β-R	GCTGTGCTGGCGGTTGTTGA
EZH2	EZH2-F	ACGGCAGCCTTGTGACAGTTC
	EZH2-R	ACACTCTCGGACAGCCAGGTAG

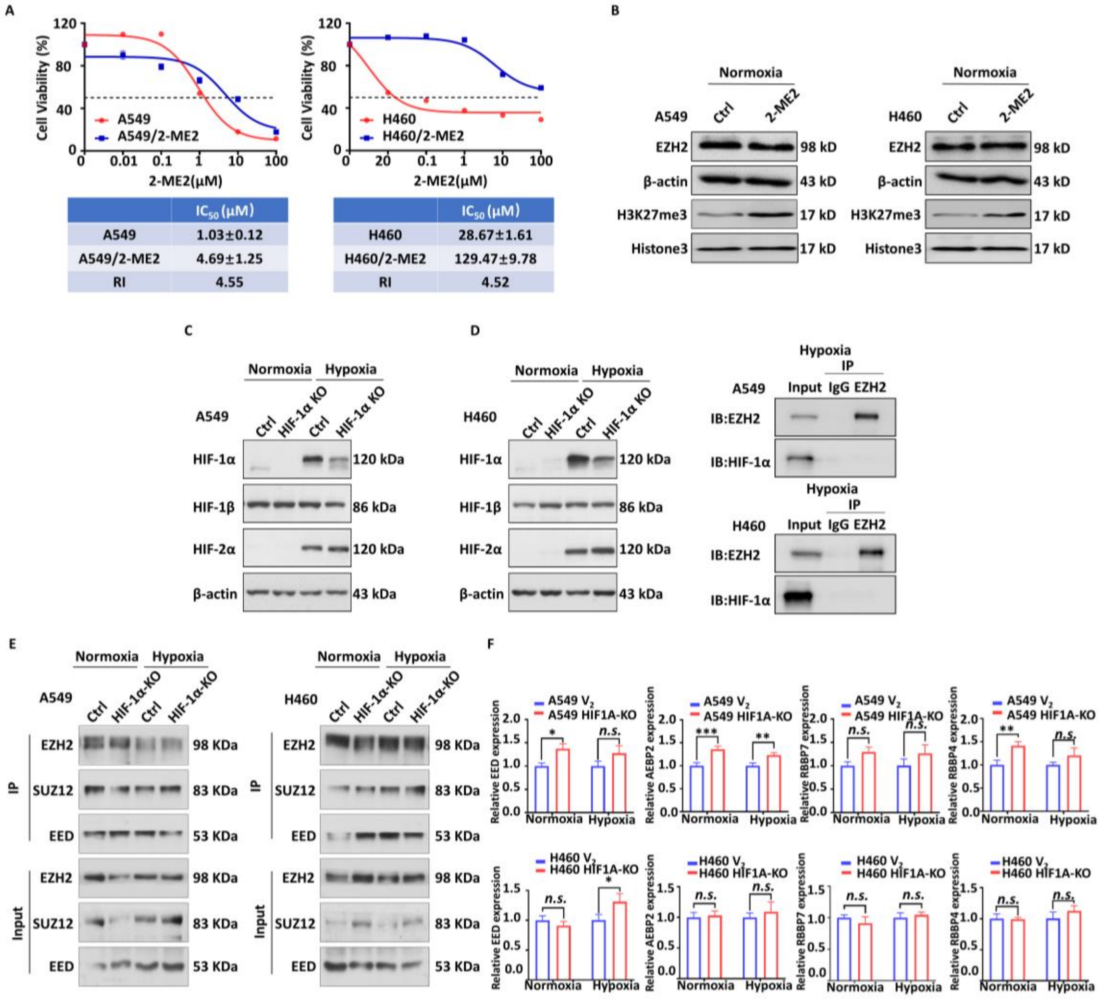

Figure S1 EZH2 mediates the resistance to HIF-1 inhibitors in lung cancer cells. (A) CCK-8 assay results show the efficacy of 2-ME2 in A549/2-ME2 and H460/2-ME2cells. Cells were treated for 72 h. (B) Expression of HIF-1α in A549/2-ME2 and H460/2-ME2 cells under normoxic conditions. (C) Knockdown of HIF-1α does not affect the expression of HIF-2α and HIF-1β. (D) Co-IP shows no interaction between EZH2 and HIF-1α in A549 and H460 cells. (E) Knockdown of HIF-1α does not promote the binding of SUZ12 and EED to EZH2 in A549 and H460 cells. (F) The expression of EED, AEBP2, RBBP4, and RBBP7 in the PRC2 complex is slightly affected by the knockdown of HIF-1α. *$p < 0.05$, as compared to the V_2 group.

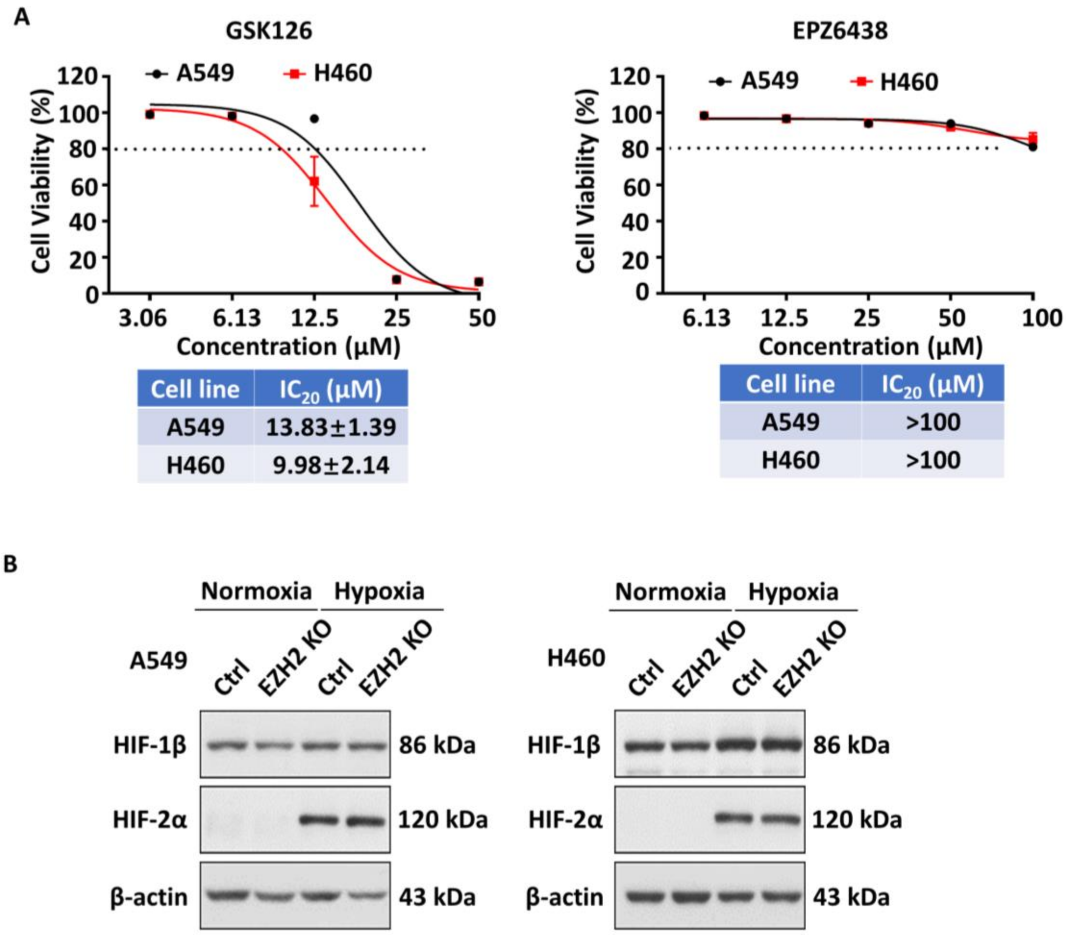

Figure S1 EZH2 regulates the expression of HIF-1α in lung cancer. (A) CCK-8 assay results show the efficacy of GSK126 and EPZ6438 in A549 and H460 cells. Cells were treated for 72 h. (B) Knockout of EZH2 does not affect the expression of HIF-2α or HIF-1β.

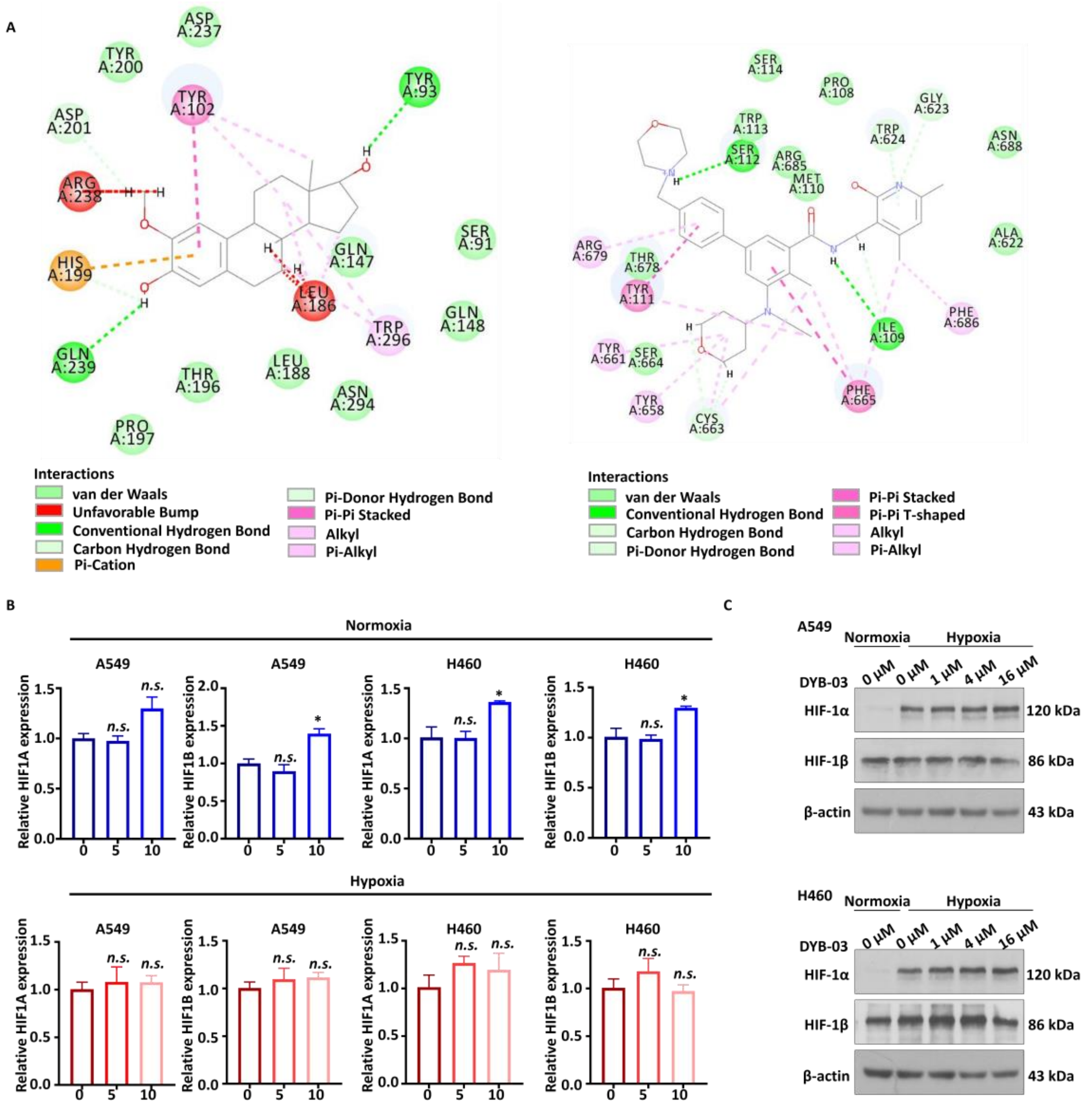

Figure S3 Synthesis, screening, and molecular mechanisms of dual-target inhibitors. (A) 2D view of 2-ME2 docked with HIF-1α (above) and EPZ6438 docked with EZH2 (below), respectively. (B) DYB-03 slightly increased HIF-1α mRNA levels under normoxia and hypoxia. (C) DYB-03 does not affect HIF-1α and HIF-1β protein levels under hypoxia.

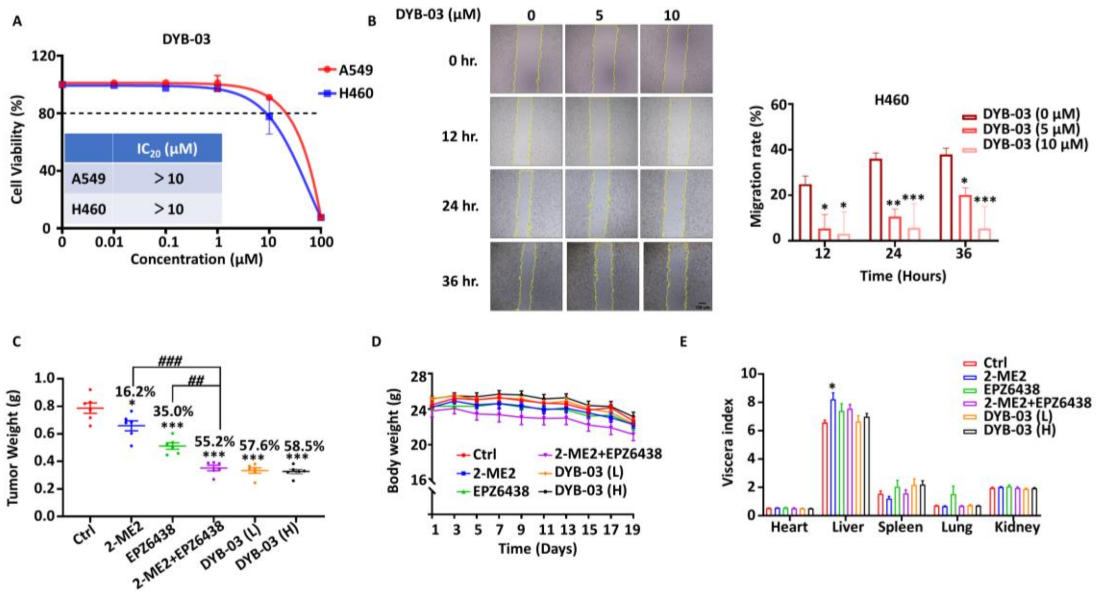

Figure S4 DYB-03 inhibits migration and invasion of NSCLC and angiogenesis *in vitro* and *in vivo*. (A) CCK-8 assay results show the efficacy of DYB-03 in A549 and H460 cells. Cells were treated for 72 h. (B) The migration of H460 cells was evaluated by wound healing assay. *$P < 0.05$, **$P < 0.01$,***$p < 0.001$, as compared to the DYB-03 (0 μM) group. (C) Tumor weights were measured in A549 xenografts treated with 2-ME2, EPZ6438, DYB-03, or the combination of 2-ME2 and EPZ6438. *$P < 0.05$, ***$p < 0.001$, compared with the control group. ##$p < 0.01$, ###$p < 0.001$, compared to the combination group. (D) Body weights and Visceral Index (E) were measured in A549 xenografts treated with 2-ME2, EPZ6438, DYB-03, or the combination of 2-ME2 and EPZ6438.

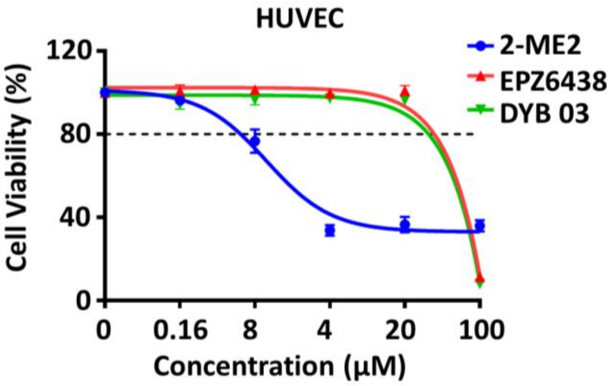

	IC_{20} (μM)
2-ME2	0.41±0.09
EPZ6438	36.37±3.53
DYB-03	14.29±2.27

Figure S5 DYB-03 inhibits angiogenesis *in vitro* and *in vivo*. (A) CCK-8 assay results show the efficacy of 2-ME2、EPZ6438 and DYB-03 in HUVEC cells. Cells were treated for 72 h.

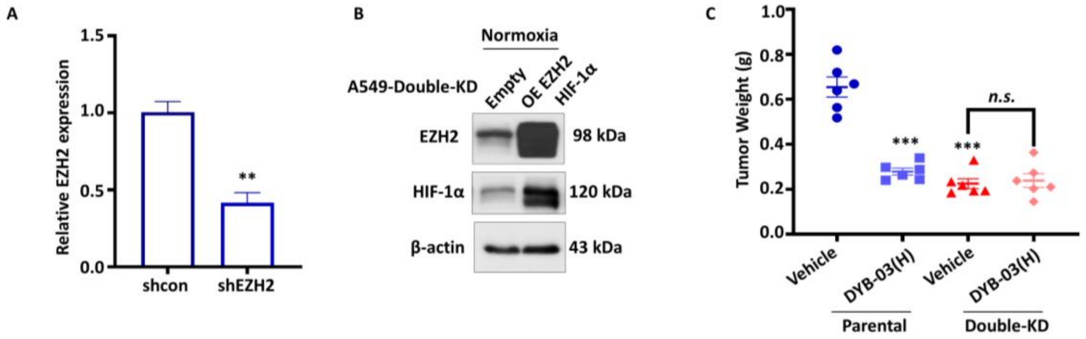

Figure S6 DYB-03 executes anti-tumor function through targeting HIF-1 and EZH2. (A) The mRNA leval of EZH2 in A549 double knockdown cells. $**P < 0.01$, compared with the control group. (B) Rescuing HIF-1α and EZH2 plasmids in a double knockdown cell line. (C) Tumor weights were measured in A549 double knockdown cell xenografts treated with DYB-03. $***p < 0.001$, compared with the control group.
